# Emergence of *Morganella morganii* subsp*. morganii* in dairy calves, China

**DOI:** 10.1038/s41426-018-0173-3

**Published:** 2018-10-24

**Authors:** Gen Li, Xudong Niu, Shiyu Yuan, Lu Liang, Yongxia Liu, Liping Hu, Jianzhu Liu, Ziqiang Cheng

**Affiliations:** 1 College of Veterinary Medicine, Shandong Agricultural University, Tai’an, 271018 PR China; 2 Research Center for Animal Disease Control Engineering Shandong Province, Shandong Agricultural University, Tai’an, 271018 PR China; 3 Animal Disease Prevention and Control Center of Shandong Provinces, Ji’nan, China

*Morganella morganii* (*M. morganii*), a gram-negative bacterium, is found in the intestinal tracts of humans and in the environment. In 1906, *M. morganii* was first isolated from a pediatric fecal culture and identified as an unimportant pathogen^1^. Since the 1970s, *M. morganii* has been considered an important cause of nosocomial infection, and some infections lead to a high mortality rate^2^. The types of diseases associated with *M. morganii* infection vary and include cellulitis, abscessation, sepsis, diarrhea, and bacteremia^3–6^.

To date, *M. morganii* infection has been reported in various animals including reptiles, elephant seals, broiler chickens, piglets, jaguars, guinea pigs, rabbits, and dolphins^7^. Notably, *M. morganii* can exist in an animal’s oral cavity and cause human infection if an animal bites or scratches a human^6,8^; thus, *M. morganii* has evolved as a zoonotic pathogenic bacteria. However, there is no evidence that *M. morganii* causes disease in cattle.

In the present study, we identified *M. morganii*-infected cattle for the first time. *M. morganii* infection resulted in a high mortality rate (57%) and severe pathological lesions. Moreover, our survey showed that *M. morganii* originated from the milk of imported cattle.

The diseased cattle were from a farm located in Tai’an, Shandong, China. The cattle farm owned 200 cattle and 100 calves, all of which were Holstein cows. In November 2017, the newborn calves showed signs of depression, poor appetite, and paralysis and excreted egg white-like feces with white flocculant material (Fig. 1a). Five days after the onset of sickness, eight of fourteen (57%) calves died, and the surviving calves showed emaciation and growth retardation. Antibiotics, such as gentamycin, sulfadiazine, penicillin, and florfenicol, were used to treat the sick cows. However, none of them were effective for treatment. Correspondingly, we conducted drug sensitivity tests of 37 drugs with the Kirby–Bauer disc diffusion method according to the National Committee for Clinical Laboratory Standards (NCCLS) regulations. The results showed that *M. morganii* was resistant to sulfadiazine, penicillin, and florfenicol and had intermediate sensitivity to gentamicin (Supplementary Table 1). These results indicated that the isolated *M. morganii* was resistant to multiple antibiotics in cattle. The autopsy results showed a large number of light yellow fibrinous suppurative clots in the abdominal cavity (Fig. 1b), gastric contents outflowing from an ulcer in the gastric fundus (Fig. 1c), white fibrinous protein on the surface of the spleen (Fig. 1d), a large amount of yellow pericardial effusion and white septic exudate in the epicardium (Fig. 1e), liver abscessation with white fibrinous purulent exudate on the surface (Fig. 1f), and white purulent material in the bladder (Fig. 1g). The lungs had connective tissue hyperplasia in the anterior lobe, edema in the posterior lobe, and a large amount of white foamy edema fluid in the trachea (Fig. 1h). The kidneys had several yellow suppurative nodules in the renal papillae (Fig. 1i).

**Fig. 1:**
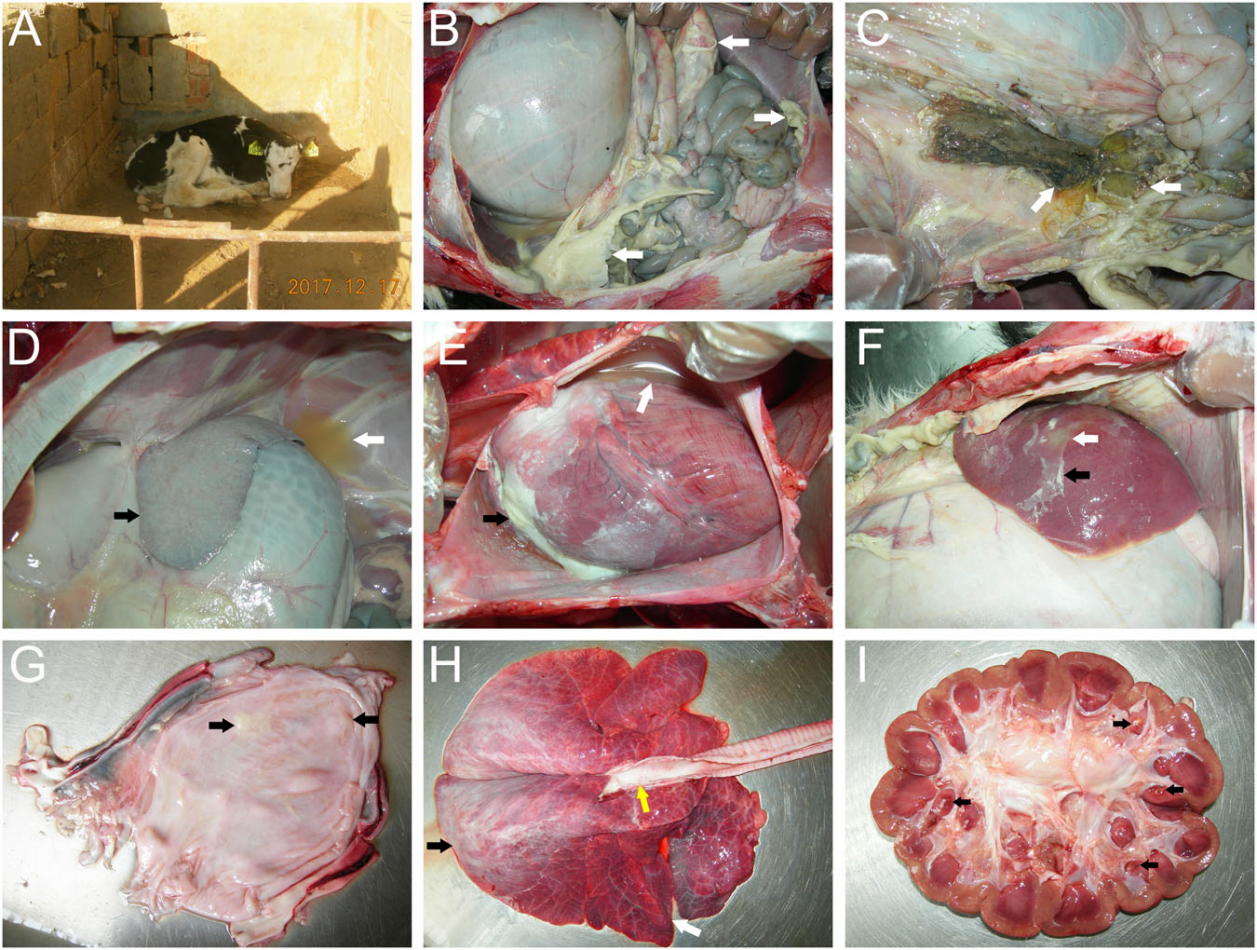
Clinical signs and pathologic lesions in a dairy calf with *M. morganii* subsp. *morganii* infection in China. **a** The newborn calves showed depression, poor appetite, and paralysis. **b** A large number of light yellow fibrinous suppurative clots in the abdominal cavity (white arrows). **c** Gastric contents outflowing from an ulcer in the gastric fundus (white arrows). **d** Yellow effusion in the abdominal cavity (white arrows) and white fibrous protein on the surface of the spleen (black arrows). **e** A large amount of yellow pericardial effusion (white arrows) and white septic exudate in the epicardium (black arrows). **f** Liver abscessation (white arrows) with white fibrinous purulent exudate (black arrows) on the surface of the liver. **g** White purulent material in the bladder (black arrows). **h** The connective tissue hyperplasia in the anterior lung lobe (white arrows), edema in the posterior lung lobe (black arrows), and a large amount of white foamy fluid in the trachea (yellow arrows). **i** Yellow suppurative nodules in the renal papillae (black arrows)

To identify which bacteria induced the infection, we sampled specimens of the infected heart, liver, spleen, lung, and kidney with an inoculation ring to inoculate tryptic soy agar (TSA), potato dextrose agar (PDA), and blood agar in an aseptic environment. Colonies grew in these three media and were gray, smooth, and round. Five colonies were selected to inoculate vegetative broth at 37 °C for 12 h with agitation, and then the bacterial DNA was extracted using the Mericon DNA Bacteria Plus Kit (Qiagen, Hilden, Germany). The bacterial 16S rDNA universal primers were used for PCR^9^. After cloning, recombinant plasmids containing the 16S rDNA inserts were sequenced by a commercial company (Huada, China). The results of the BLAST (blastn) algorithm at the National Center for Biotechnology Information (NCBI) website showed that all the five strains were *M. morganii*; we named them SDTA-1 to -5 (GenBank accession number: MH299415 to 19). By comparing the nucleotide sequence homology and analyzing the phylogenetic trees of these five strains, we found that the nucleotide sequence homology of the five strains ranged from 99.4% to 99.9% (Supplementary Figure 1), and they were present in the same branch (Supplementary Figure 2), indicating that the five strains had high homology. The genus Morganella, currently consists of a single species (*M. morganii*) with two subspecies, namely, *M. morganii* and *M. sibonii*, which can be distinguished from one another on the basis of trehalose fermentation. In addition, separation within biogroups A, B, C, D, E, F, and G is based on reactions with lysine and ornithine decarboxylases, the production of indole, and growth in the presence of KCN. After biochemical testing and identification of trehalose-negative status, the five strains were determined to belong to *M. morganii* subsp. *morganii*. Additionally, we observed that the five strains were lysine decarboxylase-negative and ornithine decarboxylase-positive and belonged to biogroup A^10^.

To explore the sources and routes of *M. morganii* infection in the calves, we sampled the cattle farm again. Samples of the drinking water, feed, soil, feces, urine, milk, and milking equipment were collected from the cattle farm for further bacterial isolation. Predictably, *M. morganii* was isolated from the milk and feces of two exotic cows. The two infected cows were introduced from a large dairy farm in Hebei Province in September 2016 and produced calves in October 2017. In the farm, milk from all the cows was collected and mixed by the breeder to feed the newborn calves. Undoubtedly, the milk from these two exotic cows was the cause of the disease. The bacterial content in the milk of these two exotic cows was 5 × 10^6^ CFU/mL, and in the mixed milk fed to the calves, the bacterial content was 7 × 10^5^ CFU/mL. We suggested that the two exotic infected cows be segregated and that the cows’ milk be boiled for 10 min before feeding it to the calves. After treatment, the conditions on the farm were controlled, and no additional calves died. In addition, considering that *M. morganii* is a pathogenic bacterium with zoonotic potential, pasteurization of raw milk will decrease the risk to the consumer.

To determine the pathogenicity of the isolated *M. morganii* strain, 40 Kunming mice were randomly divided into two groups. The infection group was administered 10^6^ CFU/mL (0.4 mL/per mouse) of *M. morganii* by gavage for 3 consecutive days, and the control group was administered normal saline solution by gavage for 3 consecutive days. Three days postinfection, the infected mice showed significant clinical symptoms, including depression and decreased feed intake (Supplementary Figure 3A). By 5 days postinfection, 14 of 20 (70%) mice had died. At necropsy, a large number of scattered white necrotic nodules were present in the liver, sporadic white necrotic foci were present on the surface of the kidneys, and hemorrhage was present in the renal pelvis (Supplementary Figure 3B–E). In addition, portions of the liver and kidney specimens were collected for repeated isolation of the *M. morganii* strain. *M. morganii* was isolated again, and no other bacteria were isolated, which conforms to Koch’s postulates. All animals used in this study were handled in strict accordance with the guidelines of the Shandong Agricultural University Animal Care and Use Committee. The approval number is SDAUA-2017-36.

Recently, with the increase in resistance of *M. morganii*, drug-resistant strains have gradually appeared, and infections caused by these drug-resistant strains often fail to resolve with clinical treatment^11^. In addition, our drug sensitivity test results also showed that *M. morganii* was resistant to multiple antibiotics. The isolated strain was sensitive to only five drugs, and we recommended using streptomycin, imipenem, aztreonam, and cefoperazone for treatment. The production of virulence factors, such as apoptosis toxin, urease, lipopolysaccharide, and hemolysin, make *M. morganii* a potential opportunistic pathogen that can cause wounds and urinary tract infections^12^. Comparative genomic analyses have shown that the pathogenicity patterns of *M. morganii* infection vary according to its virulence evolution^13^. Hosts are usually more susceptible to infection if they are immunocompromised, such as elderly, newborn, and immunodeficient patients^14,15^. In this case, the two introduced adult cows did not show any clinical symptoms or subclinical mastitis although they were carriers of *M. morganii*. However, due to the mixed feed milk, *M. morganii* was passed to the newborn calves and caused clinical symptoms and death. *M. morganii* can be isolated from the oral cavity of animals and causes infections in humans through bites or scratches^6,8^, indicating that *M. morganii* causes zoonotic infectious diseases. Therefore, milk contaminated with *M. morganii* should be considered a potential risk for transmission of infection to humans, specifically to immunocompromised hosts. Cows infected with *M. morganii* are a potentially dangerous source of this bacterium, which is highly concerning.

## Electronic supplementary material

Supplementary Figure 1

Supplementary Figure 2

Supplementary Figure 3

Supplementary materials
