# Supplementary figures and images for: Emergence of Morganella morganii subsp. morganii in dairy calves, China
Source: Emerg Microbes Infect. 2018 Oct 24;7:172. doi: 10.1038/s41426-018-0173-3 (PMC6199266; doi:10.1038/s41426-018-0173-3)

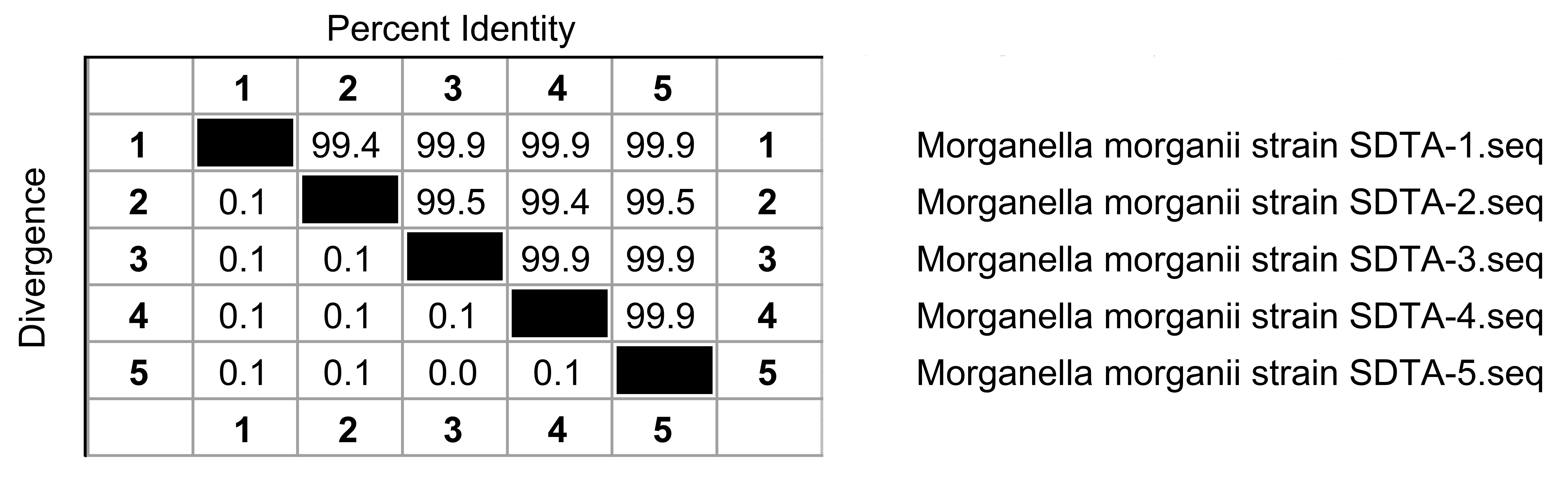

Supplement: Supplementary file 1 — Supplementary Figure 1 [file 41426_2018_173_MOESM1_ESM.jpg]

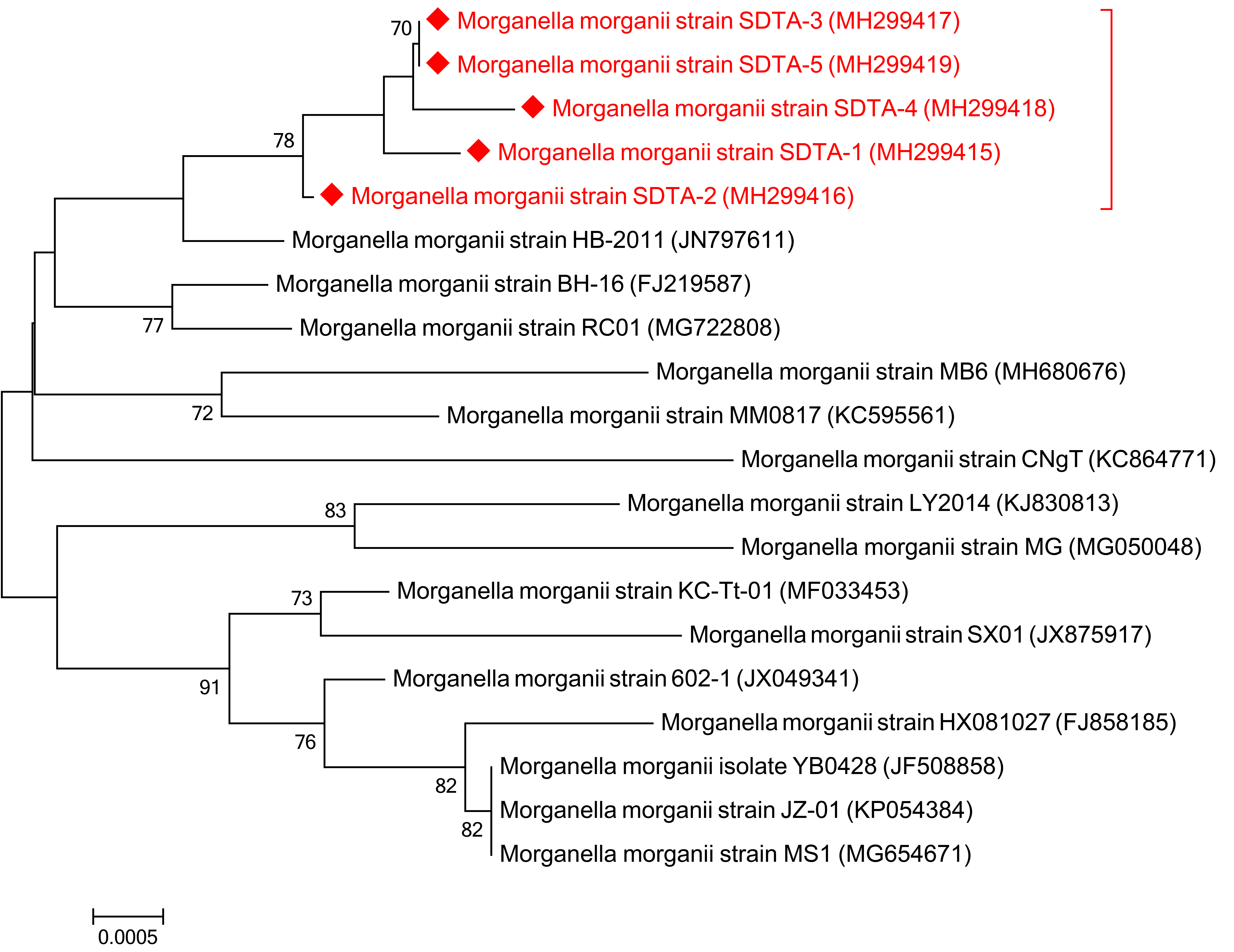

Supplement: Supplementary file 2 — Supplementary Figure 2 [file 41426_2018_173_MOESM2_ESM.jpg]

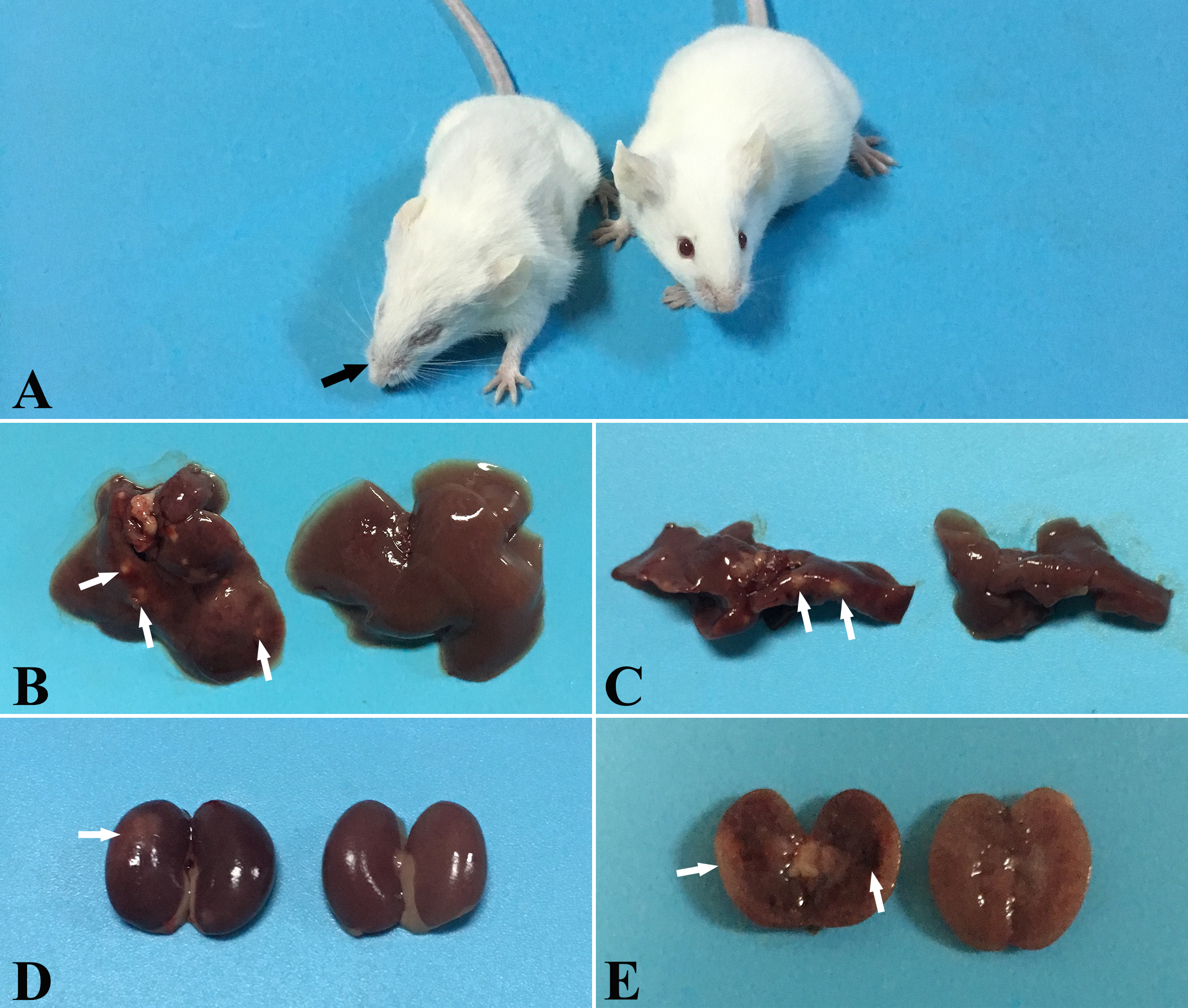

Supplement: Supplementary file 3 — Supplementary Figure 3 [file 41426_2018_173_MOESM3_ESM.jpg]
