# Supplementary material for: Emergence of Morganella morganii subsp. morganii in dairy calves, China
Source: Emerg Microbes Infect. 2018 Oct 24;7:172. doi: 10.1038/s41426-018-0173-3 (PMC6199266; doi:10.1038/s41426-018-0173-3)
Supplement: Supplementary file 4 — Supplementary materials [file 41426_2018_173_MOESM4_ESM.docx]

**Supplementary Figure 1. Homology analysis of the amino acid sequences of five *M. morganii* strains (% amino acid sequence identity).** The homology of the amino acid sequences was calculated by MegAlign software and evaluated with the Clustal W method.

**Supplementary Figure 2. Phylogenetic tree of the five *M. morganii* strains.** The five *M. morganii* strains sequenced in the study are marked with solid red squares, and the other *M. morganii* strains were found on the National Center for Biotechnology website. The phylogenetic tree was constructed with MEGA 5.1 software and evaluated with the neighbor-joining method. Bootstrapping was performed for more than 1000 replicates, and the scale bar represents 0.0005.

**Supplementary Figure 3. Clinical signs of and gross lesions in *M. morganii*-infected mice and uninfected controls. (A)** Clinical signs of the infected (left) and normal (right) mice. **(B-C)** A large number of scattered white necrotic nodules in the liver. **(D-E)** Sporadic white necrotic foci on the surface of the kidneys and hemorrhage in the renal pelvis. In each subgraph, the infected group is on the left and the normal group is on the right.

**Supplementary Table 1 Drug sensitive test**

| Name | Sensitivity | Diameter | Name | Sensitivity | Diameter |
| --- | --- | --- | --- | --- | --- |
| Amoxicillin | R | 6.5 | Furazolidone | R | 11 |
| Clarithromycin | R | 6.5 | Clindamycin | R | 11 |
| Vancomycin | R | 6.5 | Azithromycin | R | 12 |
| Sulfamethoxazole | R | 6.5 | Mezlocillin | R | 14 |
| Polymyxin | R | 6.5 | Enrofloxacin | R | 14 |
| Penicillin | R | 6.5 | Piperacillin | R | 16 |
| Ampicillin | R | 6.5 | Gentamicin | I | 13 |
| Teicoplanin | R | 6.5 | Norfloxacin | I | 13 |
| Erythromycin | R | 6.5 | Cefepime | I | 17 |
| Cephalexin | R | 6.5 | Cefoxitin | I | 17 |
| Cefradine | R | 6.5 | Fradiomycin | I | 18 |
| Oxacillin | R | 6.5 | Cefatriaxone | I | 20 |
| Lincomycin | R | 6.5 | Moxacephem | I | 20 |
| chloramphenicol | R | 7 | Streptomycin | S | 20 |
| Tetracycline | R | 7 | Ofloxacin | S | 20 |
| Doxycycline | R | 7 | Imipenem | S | 23 |
| Ceftazidime | R | 8 | Aztreonam | S | 26 |
| Florfenicol | R | 8.5 | Cefoperazone | S | 27 |
| Sulfadiazine | R | 8.5 |  |  |  |

Note: R: Resistant; I: Intermediate sensitivity; S: Sensitive; Diameter: Diameter of inhibitive tone (mm)
